# What Factors Predispose Households in Trans-Himalaya (Central Nepal) to Livestock Predation by Snow Leopards?

**DOI:** 10.3390/ani10112187

**Published:** 2020-11-23

**Authors:** Mahesh P. Tiwari, Bishnu P. Devkota, Rodney M. Jackson, Bir Bahadur Khanal Chhetri, Sistata Bagale

**Affiliations:** 1 Institute of Forestry, Tribhuvan University, Pokhara 33700, Nepal; bpdevkota@iofpc.edu.np (B.P.D.); bbkchhetri@iofpc.edu.np (B.B.K.C.); 2 Snow Leopard Conservancy, Sonoma, CA 95476, USA; rodney@snowleopardconservancy.org; 3 Department of Food and Resource Economics, University of Copenhagen, 1870 Copenhagen, Denmark; kqb949@alumni.ku.dk

**Keywords:** human-snow leopard conflict, livestock predation, Narphu valley, trans-Himalaya

## Abstract

**Simple Summary:**

Conflict between snow leopards and humans across the trans-Himalaya is a pressing conservation concern. Conflict severely impacts the socio-economy of the local pastoralist community and threatens snow leopard survival. We investigated the socio-economic factors influencing such conflict and pastoralist attitudes towards snow leopard conservation using semi-structured interviews for a better understanding of what factors influence the variability in losses among the households in the Narphu valley, Nepal. While snow leopards caused significant losses to impoverished pastoralist households, respondents were generally accepting of their presence due to religious reasons and strict law enforcement. We observed poor herding and guarding practices with households owning larger numbers of total livestock (especially large bodied animals like yak (*Bos mutus* f. *grunniens*) and cattle (*B. primigenius* f. *taurus*)), which resulted in the higher loss. Further, compensation and insurance schemes were dysfunctional, with few households insuring their livestock and receiving compensation for depredated livestock over the past two years. We recommend improving herding practices and the implementation of compensation and insurance schemes to lower the economic loss faced by herders due to predation and creating positive avenues for the co-existence of snow leopards and humans.

**Abstract:**

Livestock depredation across the trans-Himalaya causes significant economic losses to pastoralist communities. Quantification of livestock predation and the assessment of variables associated with depredation are crucial for designing effective long-term mitigation measures. We investigated the patterns and factors of livestock depredation by snow leopards (*Panthera uncia*) using semi-structured questionnaires targeting herders in the Narphu valley of the Annapurna Conservation Area, Nepal. During the two years (2017/18 and 2018/19), 73.9% of the households interviewed (*n* = 65) lost livestock to snow leopards, with an annual average loss of two livestock per household. Of the total depredation attributed to snow leopards, 55.4% were yak (mainly female: 79%), 31.7% goat, 6.8% sheep, 3.2% horse and 2.8% cattle. Results from applying Generalized Linear Mixed Models (GLMMs) revealed the total number of livestock owned and the number of larger bodied livestock species as the main explanatory covariates explaining livestock depredation. Forty-one (41%) of all herders considered snow leopard’s preference for domestic livestock as the main factor in livestock predation, whereas only 5% perceived poor herding practice as the main reason for the loss. Our study found poor and changing herding practices in the valley, whereby 71% herders reported careful herding as a solution to snow leopard depredation, and 15% of herders considered the complete extermination of snow leopards as the best solution to the problem. Tolerance levels and awareness among herders towards snow leopard conservation is increasing, mainly due to the Buddhist religion and strict law enforcement within this protected area. We recommend the effective implementation of a community-based livestock insurance scheme to compensate the economic loss of herders due to predation and improved herding practices as the recommended mitigation measures for ensuring livestock security and snow leopards’ conservation in the valley.

## 1. Introduction

Top predators such as snow leopard (*Panthera uncia*) and Himalayan wolf (*Canis lupus chanco*) are key mountain species across Himalayan range countries (i.e., Bhutan, China, India and Nepal) with their presence indicative of a healthy ecosystem [1]. However, these species are increasingly entering villages and pastures due to lack of wild prey as well as habitat fragmentation and degradation. The environmental outcomes associated with anthropogenic activities disrupt ecosystem integrity and their functions. Such factors cause conflict with the local communities where both humans and top predators share common resources to meet their survival needs [1,2,3]. Human conflicts with carnivores mainly occur in the form of livestock depredation, and sometimes tragic consequences of human injury or death [4]. Livestock predation is the most common conflict type observed with snow leopards and wolves [5].

In Nepal’s Himalaya, 300–400 snow leopards reside across three habitat blocks viz. Western, Central and Eastern landscapes [6]. These landscapes contribute to one of the critical habitat linkages of snow leopards in the Tianshan-Pamir-Hindu Kush-Karakorum region where their population is facing threats from poaching, anthropogenic development and climate change [7,8]. These landscapes are inhabited by typically marginalized impoverished pastoralists, who are highly reliant upon herding for their subsistence and income. Pastoralists lose livestock due to disease and other natural causes, along with predation by snow leopards. Predation fosters intolerance among such impoverished pastoralists and often leads to persecution [5,9,10]. The conflict between humans and snow leopards is reported across snow leopard range countries, causing significant economic losses to local pastoralists, thereby creating a strong dislike towards the species and decreased support for its conservation [11,12].

Livestock losses due to predation in Nepal are reportedly high but vary widely in terms of magnitude [2,3,13]. As a result, conservation initiatives can cause tense relationships between park officials and local people, whereby pastoralists act in a polemical manner against park officials for prioritizing conservation over the severe socio-economic consequences caused by predation [14]. Some local community members view complete extermination of the species as the only solution to safeguard their livelihood [13]. In this regard, community-based conservation initiatives have been recognized for producing promising results by including pastoralists in wildlife protection [15,16].

The pattern and intensity of livestock losses by predation varies spatially; losses appear lower at higher spatial scales, such as in a landscape and higher when quantified at smaller scales, such as the village level [2,12]. Chetri et al. [17] found a similar pattern of livestock depredation by snow leopard in the Annapurna-Manaslu landscape of Central Nepal, where annual depredation rate was less than 2%. On the other hand, Oli et al. [13] in lower Manang and Aryal et al. [3] in upper Mustang of the Annapurna Conservation Area Project (ACAP) observed annual depredation rate greater than 2%. Similarly, previous snow leopards diet analysis using a microhistology approach with scats from Phu valley in Annapurna Conservation Area revealed an annual livestock depredation rate of 4.0% [18], which is high despite the availability of wild prey species in this region [19]. Assessment of factors that could explain this unpredictable pattern and varying levels of losses within a landscape is needed [17,20]. However, herding patterns and complexity of associated socio-economic factors vary within a landscape and from one village to another [12]. It is, therefore, necessary to closely examine losses on a site-specific basis (e.g., village) when designing practical field-based solutions.

Questionnaire interviews provide valuable information, including determining the presence/absence of wildlife species, as well as attitudes and perceptions of local communities towards wildlife conservation at a local level [21]. Thus, this study used questionnaire interviews to fill current data gaps surroundings depredation in the Narphu valley, incorporating predation data from both the villages (Nar and Phu) and to determine if the predation has increased or decreased in recent years. We further aimed to assess possible changes in herding practices and evaluate factors that may help explain such variability in losses among the pastoralist’s households. The primary research questions of this work were: Do some households lose more livestock to snow leopard than others? Why? The study thus investigated local context and events, which are vital in designing practical field-based solutions toward establishing positive avenues of coexistence between snow leopards and humans.

## 2. Materials and Methods

### 2.1. Study Area

**Figure 1:**
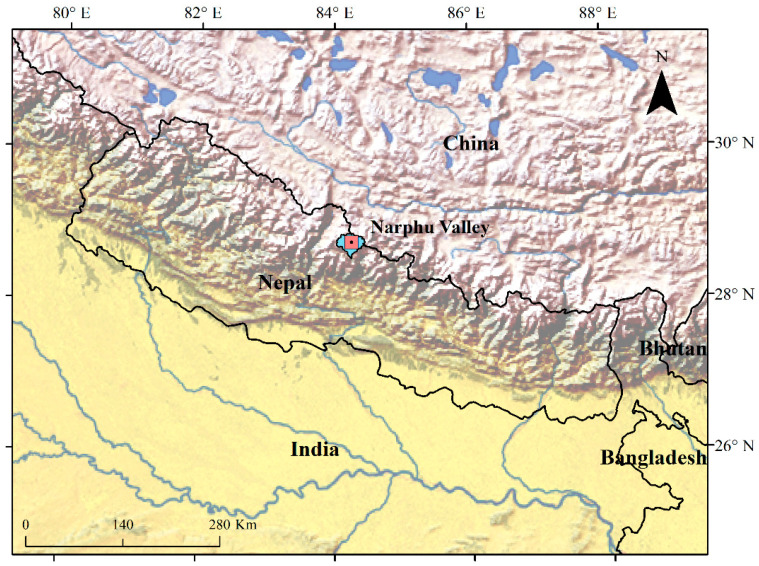
Map showing the location of study area, Narphu valley, Gandaki Province, Nepal.

Narphu valley (28° 42′ E Latitude and 84° 9′ N Longitude) lies in the Manang district of Gandaki Province, Nepal (Figure 1). The valley extends over an area of 838 km^2^ with elevations ranging from 3700 m to 6000 m above mean sea level [19]. The valley possesses scant human settlement: Phu village in the north extends to the Tibetan Plateau while Nar Village is located in the south. According to the most recent national census (2011), the total population of the Narphu valley is 554 persons comprised of in 122 households [22]. Narphu valley lies within the rain shadow of the Annapurna Himalaya with the topography dominated by steep slopes, massive cliffs and glaciers. It is semi-arid with total annual precipitation of less than 400 mm, usually in the form of snow. Vegetation cover includes alpine meadows in valley basins, scrubland along rugged and dry southern slopes and alpine grassland at higher altitudes within the northern regions. Blue sheep (*Pseudois nayaur*) are the only wild ungulate species, while snow leopard (*Panthera uncia*) and red fox (*Vulpes vulpes*) are the primary carnivores. The presence of Himalayan wolf (*Canis lupus chanco*) and brown bear (*Ursus arctos*) in this location have not been recorded [19]. Animal husbandry represents the main occupation of the local pastoralist community. In addition to herding, people cultivate crops (potato and barley) in the summer. Tourism has yet to take a foothold here due to its very remote and risky trekking trails.

Like in other parts of the trans-Himalaya, livestock in Narphu valley are kept far from the village at higher pastures during summer months (June–August) [19]. While small bodied livestock (sheep and goats) are often closely attended, larger bodied livestock (yak and horse) are scattered and left unattended in pastures. However, the herds of yak and horse are periodically checked and are given a salt-water solution to drink. In winter (December–February), livestock including yak, are taken down to lower elevations, at and below the villages. Although small bodied livestock are stall fed, larger bodied livestock graze in lower pastures close to the settlements. During winter, yak of some households are merged and are mainly herded by older people whereas the youths move to nearby towns and Nepal’s capital for trading [23]. Spring (March–May) marks the calving period of yak and the beginning of the agriculture season (ploughing fields and planting crops). Thus, few herders stay on the pasture for milking while others return to the village with male yak to work on the fields. With the onset of autumn (early September), herders descend to the village to celebrate *Dardze* (a local festival) and livestock are kept close to settlements. In October and November, crops such as potatoes, buckwheat and barley are harvested, and livestock are allowed to graze upon the stubble of harvested fields [23].

### 2.2. Questionnaire Survey

The study was conducted in accordance with the Declaration of the Helsinki and the protocol approved by the Ethics Committee of Department of National Parks and Wildlife Conservation, Nepal (Research Permit ID: 169/1950 - 075/76 and permit approved date: 24 February 2019).

Households (*n* = 65) were surveyed between March 2019 and April 2019 in Narphu valley to document livestock losses over the previous two years (2017/18 and 2018/19). Because we surveyed the households towards the end of winter (or the beginning of spring), most of them were still in the process of returning to the village. In winter, only a few herders remain in the pasture, whereas others move down to lower settlement at or below the village in order to avoid the harsh winter [23]. Thus, we interviewed all the households that were available during our survey period to increase our sample size.

The two villages (Nar and Phu) of the valley are located a day’s walk apart on foot; houses are arranged in each village in clusters, saving considerable time walking from one household to another during the survey period. We interviewed the head of each household sampled. When the head of the family was not available, we interviewed the most senior member of the family. Prior to the interview, an ethical consideration was requested and we stated that the respondent might leave the interview if they felt uncomfortable or could not participate further in the interview. Further, we made them aware of the study’s purpose and confirmed that all personal information would be kept confidential [24]. We used pre-determined questionnaires (File S1) to record details of livestock loss for assessing the drivers of predation and local attitudes towards snow leopards and their conservation. To understand the extent, pattern and drivers of livestock depredation, we asked respondents about the number, type and cause of each livestock loss, each event’s date, time and place of attack/loss due to snow leopards, as well as the herding practices and mitigation measures for preventing or reducing predation attacks on livestock.

For analyzing livestock data, we coded a 0.5 for a young individual of each livestock type and a 1 for an adult individual of each livestock type. We summed these coded numbers to reach the number of livestock. We used this method because respondents were not always correctly able to remember livestock age classes [25], but they were better able to classify them as young or adult. In addition, for computing economic losses, we used the average market price value of each livestock type, which could provide a more representative loss if two young animals were counted as one. We asked local herders, traders and the president of Annapurna Conservation Area Snow Leopard Conservation Committee (SLCC) to estimate the average price of each livestock type during the survey period, which we used to estimate economic losses caused by snow leopards. Because we conducted the interviews during the year in which the valley received heavy snowfall, we suspected that the respondent might exaggerate losses in hopes of receiving compensation. To circumvent this potential source of bias, we crosschecked reports with fellow neighbors and the local translator [2,3]. We found that the village compensation records were old and not updated and thus, we could not tally our data. The attitudes of the herders were recorded based on questions with specific anticipated answers (File S1) prepared following Suryawanshi et al. [20]. We asked seven specific questions with possible answers for each of the questions where each answer scored between −2 and +2 (Likert Scale). The total sum of these scores ranged from −9 to +9.

### 2.3. Data Analysis

We used Generalized Linear Mixed Models (GLMMs) to examine variables associated with livestock loss. Predictor variables included were: (i) The number of livestock owned, (ii) number of large bodied livestock owned, (iii) days livestock housed in pastures, (iv) family size, (v) education status of the household, and (vi) number of corrals. For determining the education status of the households, we classified the education level of each household member into three categories viz. (i) basic level (passed 8th standard), (ii) intermediate level (passed 10th standard), and (iii) higher level (passed 12th standard). We regarded the household as educated if any one of the family members fell under one of these three categories, otherwise the household was considered uneducated. Recording livestock number is count data, though we used decimal responses as well. Thus, we rounded up the response variable up or down before fitting the models.

All variables were entered into Excel and imported to R Version 4.0.0 [26] for statistical analyses and modelling. Our non-normal count data showed over-dispersion with some zero’s (household losing no livestock), therefore we used negative binomial regression models over the Poisson model [27]. Prior to model fitting, predictors were scaled and checked for multi-collinearity using Pearson’s correlation coefficients; variables with |r| > 0.7 were not used in the same model [28]. For example, the variable number of corrals and the total number of livestock owned by each household (*r* = 0.75) was not used in the same model. We fitted 18 candidate models and used Akaike’s Information Criterion (AICc), corrected for small samples to rank the models [29]. Predictor factors were used as the fixed effects on each model while the village was made a random effect. We used model averaging from the MuMIN package in R to calculate the weighted average of parameter estimates and the relative importance of each set of predictors by summing the AICc weights of each predictor set [30]. Models where the delta AICc was <7 were included in model averaging. With the Chi-square test, we tested if seasons and livestock predation by snow leopards were related and if the proportion of livestock predation were related to livestock species. In addition, we used Wilcoxon tests between livestock predation loss by snow leopards for all possible season pairs.

## 3. Results

### 3.1. Livelihood and Herding Practices

Major economic activities were agriculture/livestock aided by Yarshagumba (*Ophiocordyceps sinesis*) collection, while several households received remittance and a few were novice hoteliers (lodge owner-operators). The total number of livestock recorded was 2149 individuals in 65 households surveyed. Among these households, yak (*Bos mutus* f. *grunniens*, 51%) were the most abundant livestock followed by goats (*Capra aegagrus* f. *hircus*, 22%), sheep (*Ovies orientalis* f. *aries*, 14%), horses (*Equus ferus* f. *caballus*, 7%) and taurine cattle (*Bos primigenius* f. *taurus*, 6%). The average herd size per household was 33.06 (SE 3.35), with a range of 1–116. The average holding of yaks per household was 17 with the range 0–60 whereas the average holding of small bodied livestock (sheep and goats) were 12 with the range of 0–87. Horses (average 2 individuals) were kept as a means of transportation, mainly to carry goods from the district headquarters.

Ninety two percent of the herders reported a decreasing trend of livestock numbers over the previous two years. This decrease was attributed to four reasons: (i) Predation by snow leopards (50% of the households) (ii) death of livestock due to natural disasters, starvation, accidents and poor pasturage, 49% (iii) lack of human resources, 22%—older herders are mostly involved in herding practices while the youths move out of the valley for education or vocation purposes (iv) alternative income sources such as tea house trek hoteliers, Yarshagumba collection and remittance, 5%. An older herder, aged 70 shared

“I am old and too weak to guard the livestock, which is the major reason for predation. Therefore, the number of livestock has also lowered compared to past years. The predation rate may be decreased if we are provided with raincoats, cloth for scare crows, and lights”.

Only two herders (owning 116 and 60 livestock, respectively) reported an increase in livestock, and both herder households whose income sources were fully dependent on agriculture/livestock and Yarshagumba collection.

Herders tend to move an average of 3 times per year for yak grazing and often attributed this movement to low/insufficient forage in pastures. We observed that each herder encountered live snow leopards 1.6 times per year on average during 2017/18 and 2018/19. Kyang and Namgya are the two major summer pastures for grazing livestock, suggesting a dearth of other productive pastures in this region. The average number of days that large bodied livestock were grazed in the pasture was 199 days. Nearly two-third (72%) of herders self-herded their livestock, whereas 17% reported that they herded in collective groups. Corrals in the valley were mostly traditional in construction made with stones (75%). A few herders reported possession of predator proof corrals (18%) and 9% owned both types.

### 3.2. Livestock Losses and Predation by Snow Leopard

Based on the interviews (*n* = 65 households), 420.5 livestock were lost over the past two years; 126 small bodied livestock and 294.5 larger bodied livestock, wherein snow leopards predation accounted for 92 and 150.5 losses, respectively (Table 1). Predation by snow leopards was the major speculated cause for livestock loss, accounting for 58% of all incidents or an average annual loss of 2 livestock per household. Additionally, a single incident of mass killing was reported over the last two years, whereby one household lost 37 goats to one snow leopard in a single night. Mass killing of livestock also occurs in other carnivores such as wolves (*Canis lupus*). One goat was reported to be taken by a golden jackal (*Canis aureus*, 0.24%; Table 1). The majority of snow leopard losses occurred predominantly during the night (38%), followed by daytime (28%), evening (19%) and morning (15%). The proportion of livestock predation by snow leopard for different livestock species was significantly different (*χ^2^* = 269.32, *df* = 4, *p* < 0.001) (Table 1). The average annual depredation rate due to snow leopard predation on livestock was 5.6%. Despite a higher predation by snow leopards, only two herders received compensation. One of the herders expressed his strong dislike exclaiming:

“It is better if we could fresh locate the dead livestock, at least we could consume it. Although the compensation policy is appreciating, it is very challenging to get the photographs of dead livestock and even more expensive to claim money since it involves long distance travel to ACAP office where the expense exceeds our compensation amount”.

**Figure 2:**
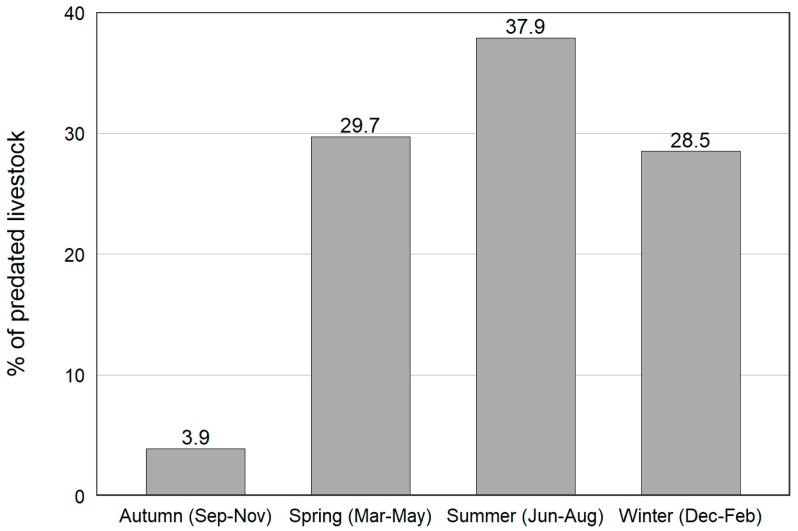
Depredation of livestock by snow leopards across seasons among surveyed households (*n* = 65) during years 2017/18 and 2018/19.

Livestock depredation was highest during summer with significant seasonal differences in depredation (*χ^2^* = 25.82, *df* = 3, *p* < 0.001) (Figure 2). However, autumn-spring, autumn-summer and autumn-winter seasons were significantly different (*p* < 0.001) whereas Wilcoxon tests showed no significant difference between spring-summer, spring-winter and summer-winter season pairs. The majority of the predation events occurred in remote pastures (156.5 cases, 65%) when no human guards were present to look after the herd. More than half (51%) of the depredation events occurred in pastures Namgya and Kyang. Most of the yak (13% of total herd) killed by snow leopards were females (107.5 individuals out of 136.5, 79%) which were attacked predominately during summer when grazing in higher pastures and spring, coinciding with the lambing period and the presumably weaker state of females.

The average annual financial loss due to snow leopard among the sampled households was $270.56 USD per households with the greatest loss from yak ($29,934 USD, Table 2) for the two-year period.

### 3.3. Factors Responsible for Livestock Depredation

We observed that the loss of livestock among households was best explained by the interaction effect of total number of livestock and number of larger bodied livestock owned (Table 3). No single model best supported our data (AICc weight greater than 0.9 indicate the best fit model). The variables, number of livestock (relative importance, 0.98) and number of larger bodied livestock (relative importance = 0.82) significantly influenced the predation by snow leopard (Table 4). No other variables explained losses among the households. Although not significant, it was observed that uneducated household tend to lose more livestock (*p* = 0.72, Table 3 and Table 4). While the observed SE for variables CN (number of corrals) and DP (days livestock housed in pastures) were slightly larger than the estimate (Table 4), we used Pearson’s product-moment correlation test to examine their degree of association with the response variable. We found significant positive correlation between loss and number of days herding in pastures (Pearson’s product-moment correlation coefficient, *r* = 0.36, *df* = 63, *p* = 0.003), family size (*r* = 0.28, *df* = 63, *p* = 0.021) and corral number (*r* = 0.39, *df* = 63, *p* = 0.001).

### 3.4. Attitude towards Snow Leopard Conservation

The overall mean attitude score towards snow leopard conservation was 0.08 (Table 5). We found variable response in attitudes of herders towards snow leopard conservation. A total of 49% of the herders held negative, 45% held positive and 6% held neutral attitudes in the final total scoring (Table 5). We observed the Buddhist religion and strict law enforcement to be strong influencers of attitudes. For example, one woman reported

“When we kill Pange (snow leopard), the mountain god gets annoyed and we receive more losses and natural disasters in the future. This has happened in the past too”. However, a resident aged 25 responded “Establishment of ACAP office have forbidden us to shoot the species. Snow leopard causes us problem, if allowed or lifted the enforced law, people might shoot the species”.

We observed positive attitude of herders for questions regarding conservation education and protection of snow leopards whereas negative attitudes for environmental benefit of snow leopard’s presence and reaction to livestock predation by snow leopard (Table 5).

Regarding measures to reduce losses and minimize conflicts, 71% of the herders reported careful herding practices, 15% suggested complete extermination of the species, 3% suggested avoiding predation risk areas while herding livestock, and 4% of herders were undecided about which method would be appropriate for minimizing conflicts with snow leopards.

## 4. Discussion

This study increased our understanding of conflicts between humans and snow leopards in one of the least explored parts of the Annapurna Conservation Area, Nepal. Our study found high reported levels of predation of livestock by snow leopards compared to previous studies from other parts of Annapurna Conservation Area [3,13,17,18]. However, the rate of livestock predation was somewhat consistent with some other parts of the trans-Himalaya region [31,32,33,34,35]. The high number of livestock losses to snow leopards may be attributed to the fact that it is the only top carnivore predator recorded in the valley. Genetic analysis of snow leopards scats estimated the presence of 6 snow leopards in vicinity of Phu village of the Narphu valley, implying a high population density [18]. Higher predator density implies greater probability of encounters with domestic animals in pastures being more vulnerable to attack [36]. The aggregative response of snow leopards, despite an abundant natural prey base, may help explain such a high loss in the valley [18,37]. While conservation initiatives and promotion of eco-tourism in protected areas increase wildlife numbers, their restricted distribution within fragmented habitats inside protected areas might also locally intensify conflicts with humans [38]. Our estimated monetary loss due to snow leopard predation (US $270.56 per household) is slightly greater than the adjoining lower Manang area. Oli et al. [13] estimated an average annual loss of 25% of the mean average per capita income of Nepal in lower Manang.

Higher predation rates and decreasing mobility of older herders has forced them to reduce their livestock holdings (33.0 in our case vs 53.0 in previous studies), along with encouraging yak ownership over smaller livestock [18,39]. We found poor and changing herding practices, with traditional preventive methods (e.g., blowing yak’s horn, fire, cloth scarecrows, shouting and throwing stones) becoming largely ineffective to preventing predation. Moreover, we observed only two guard dogs in the valley. They were used to guard the monastery (*Gumbas*) rather than livestock at night in corrals or while grazing in the pastures. Lax herding practices and lack of predator-proofed corrals contribute to overall livestock depredation [2,5]. We speculate that other causes in the decline of livestock ownership may result from alternative income sources like tea house trekking tourism, outside-job remittances and scarcity of herders for tending the larger yak holdings. Herders are mostly old with the younger generation (especially males) migrating to urban areas in search of better education and employment opportunities. Implementation of community-based conservation approaches such as Snow Leopard Conservation Committees (SLCCs) in Nepal has been established for implementing community-based livestock insurance schemes and conservation of snow leopards in specific localities [15]. However, the poor functioning of SLCCs and implementation of community-based insurance scheme—only six households with livestock insurance in this study—stand as a major challenge for efforts aimed at creating the peaceful co-existence of humans and snow leopards—especially where functionality of such an approach is insufficiently monitored due to the remoteness of this valley. Moreover, the expenses incurred while filing compensation claim exceeds the loss payment. Herders expressed dislike for the tedious procedure involved in requesting and for receiving compensation.

Yak, in Phu valley, were observed grazing in the high pastures overlapping the distribution of the only large prey species (blue sheep), whereas small bodied livestock tended to graze in lower pastures closer to the settlement and often with shepherd guarding [19]. Given the herding practices in autumn, and the habitat overlap between yak and blue sheep, we observed low predation in autumn and suspect snow leopards during this season predominately consumed wild prey (i.e., blue sheep). In the Phu valley and across ACAP, livestock was previously found to contribute about 42% of the snow leopards diet [18], whereas Devkota et al. [40] found no proportional relationship between wild prey density and snow leopard diet. Scat analysis also revealed yaks to be the most commonly present domestic livestock species in snow leopard scats from upper Manang [41]. We suspect that snow leopard prey preference may have changed or alternatively that the wild prey-base may have declined in recent years, leading to the greater dependence on yak.

Size of the livestock holdings and larger bodied livestock were found to be major covariates explaining why some households experienced higher loss than others. Households owning more livestock (>30) had 89% of the depredation recorded whereas households owning fewer livestock (<10) lost 6% and households with normal numbers of livestock (10–20 individuals) lost 10% of the livestock depredation. Our result showed high predation of yaks and goats, which may be due to the larger herd size compared to other types of livestock (Table 1) [17]. Thus, indicating the need of further studies to examine if the herd size has an effect on the survival of the different livestock species. In our case, we observed a greater number of large stock (which graze in scattered patterns; are difficult to guard) than small stock (typically graze in a group; easy to guard), thus increasing the risk of depredation [17,42]. Similar observations were documented by other investigators in other parts of the Annapurna Conservation Area and the Shey-Phoksundo National Park, Nepal [17,40]. Thus, regulating livestock composition (livestock species and their numbers) could help reduce the depredation losses [17,42]. For example, modifying livestock composition through increasing smaller livestock (sheep and goats) and reducing the number of larger free-ranging livestock would help minimize the loss. Moreover, within in large bodied livestock like yak in our case, reducing the number of female yaks where a female could mate with one or a few males and maintain productivity could further reduce the loss and subdue economic loss of the herders. Although not significant, the direction of the variable—number of days in pasture—was positive, with depredation increasing with increase in number of herding days in pastures and herders not being sufficiently vigilant. While older herders may be more reliable at guarding than the younger generation who are less likely to take up their profession, we observed greater losses among uneducated households confirming the need of capacitating older herders on careful herding techniques. This also suggests gaps in knowledge transfer between educated young offspring and the older parents (herders). Most of the outreach and conservation initiatives are designed to involve students and youths, although they are little involved in herding livestock.

The total mean attitude scores of respondents close to zero suggests that local people were very weakly accepting the presence of snow leopards, probably due to the high level of livestock losses by snow leopard [20]. On the one hand, people were accepting the species due to their religious belief (Buddhist) and strict law enforcement for snow leopard conservation, whereas on the other hand they feared livestock loss from the species [43]. We also observed that herders responded negatively on the presence of snow leopard benefitting the environment. They often misattribute their livestock loss as the fault of snow leopard’s presence while perceiving its presence as being environmentally detrimental due to lack of education (Table 5). People’s support for education can also be linked to their limited local access to the formal education system which forces them to migrate to urban areas for educational purposes. Further, their negative response when asked about how would they react to predation by snow leopard confirms the need for conservation education and outreach toward encouraging positive attitudes and environmental stewardship by local people (Table 5). The herders’ positivity in supporting ACAP for snow leopard conservation could be attributed to strict law enforcement, their Buddhist religion and increasingly positive attitudes toward conservation [25,43,44]. However, the escalation of conflicts and risk of retaliatory killing of snow leopards cannot be ignored [45].

## 5. Conclusions

We found a decrease in livestock holdings with higher predation from snow leopards over the study period. Herders owning larger livestock size and larger bodied livestock incurred more economic loss due to livestock depredation. The livestock herding trend is decreasing mainly due to livestock predation by snow leopards and outmigration of village youths to urban centers. In order to reduce economic losses, we recommend encouraging small bodied livestock (with proper guarding, assuming sufficient household members for shepherding) while encouraging other appropriate changes in livestock composition (number and livestock species). Moreover, the effective implementation of a community-managed insurance program, with education toward improvement of herding practices and equitable compensation for depredation losses would help in building positive attitudes among herders for supporting snow leopard conservation and minimize these conflicts in the Narphu valley. Similarly, conservation programs focused on empowering herders through educational programs to improve herding techniques and herder motivations (mainly older pastoralists) would likely reduce overall losses, especially when livestock are grazed on open pastures or housed in poorly constructed corrals at night. Programs that reduce out-migration of the younger generation and encourage them towards herding could help preserve traditional herding practices in the Narphu valley. For reasons stated above, this paper highlights conflict between snow leopard and humans as a complex phenomenon requiring holistic, multi-pronged approaches for minimizing negative impacts and ensuring positive species coexistence with pastoral-dependent communities.

## Figures and Tables

**Table 1:** Livestock holdings and loss among sampled households (*n* = 65) in Narphu valley during years 2017/18 and 2018/19.

Livestock	Livestock Holding	Causes of Livestock Loss						
		Natural Disaster	Disease	Starvation	Accident	Snow Leopard	Others	Loss Total
Goat	481	−	6.5	13.5	−	76.5	1	97.5
Sheep	292	3	2	8	−	15.5	−	28.5
Cattle	129	2	−	12	1	6.5	−	21.5
Yak	1096	112	1	4	2.5	136.5	−	256
Horse	151	2.5	1	2.5	3.5	7.5	−	17
Total	2149	119.5	10.5	40	7	242.5	1	420.5
Loss %		28.42	2.50	9.51	1.66	57.67	0.24	

**Table 2:** Estimated economic loss due to snow leopards for the sampled households (*n* = 65) for years 2017/18 and 2018/19.

Livestock Types	Number of Predated Livestock	Average Market Price (NPR.)	Total (in USD *)
Goat	76.5	3500	2349
Sheep	15.5	3800	517
Cattle	6.5	7000	399
Yak	136.5	25,000	29,934
Horse	7.5	30,000	1973
Total	242.5		35,172

* 1 USD = Nepalese Rupees (NPR). 114 (Currency exchange rates of USD and NPR. during the survey period).

**Table 3:** Results from Generalized Linear Mixed Models (GLMMs) for livestock loss due to snow leopards. TL = total number of livestock owned, LS = number of larger bodied livestock owned, DP = days livestock housed in pastures, CN = number of corrals, FS = family size and EDU = education status of the households. Here, only those models with Akaike’s Information Criterion (AICc) weights greater than 0.01 are presented (see Supplementary Materials, Table S1 for full models).

Model	df	logLik	AICc	Delta	Weight
TL × LS	6	−140.04	293.52	0.00	0.32
TL × LS + FS	7	−139.59	295.15	1.63	0.14
TL × LS + EDU	7	−139.97	295.90	2.37	0.10
TL × LS + CN	7	−140.04	296.04	2.51	0.09
TL × LS + DP	7	−140.04	296.04	2.51	0.09
TL	4	−143.82	296.32	2.79	0.08
TL + FS	5	−142.91	296.84	3.32	0.06
TL + LS	5	−143.31	297.63	4.11	0.04
TL + EDU	5	−143.77	298.57	5.04	0.03
TL × LS + DP	8	−140.03	298.64	5.12	0.02
TL + LS +DP	6	−142.75	298.95	5.43	0.02
TL + LS + DP + FS	7	−141.95	299.87	6.34	0.01
Intercept only	3	−154.66	315.71	22.19	0.00

**Table 4:** Model averaging and results from 95% confidence interval for livestock loss due to snow leopards; Relative importance (sum AICc) of the predictors were obtained for models whose delta AICc was less than 7. TL = total number of livestock owned, LS = number of larger bodied livestock owned, DP = days livestock housed in pastures, CN = number of corrals, FS = family size and EDU = education status of the households.

Predictors	Estimate	Adj. SE	Z value	Pr(>|z|)	Sum AICc
TL	0.51	0.13	3.75	0.000 **	0.98
LS	0.37	0.18	2.10	0.035 *	0.82
FS	0.14	0.13	1.06	0.288	0.20
EDU (uneducated)	0.11	0.30	0.36	0.719	0.12
CN	−0.01	0.19	0.07	0.946	0.11
DP	0.01	0.19	0.05	0.958	0.14

** *p* < 0.001 and * *p* < 0.05.

**Table 5:** Scoring from attitude survey of herders (*n* = 65) towards snow leopard conservation ^1^. (ACAP = Annapurna Conservation Area Project).

QN	Questions	No of Respondents Reporting (*n* = 65)				
		Positive (> 0 ≤ 9)	Neutral (Score 0)	Negative (< 0 ≥ −9)	Total Score	Mean Score
1	Do you always want to see snow leopards in Narphu valley?	25	15	25	0	0.00
2	Do you think snow leopards should be conserved?	30	15	20	10	0.15
3	Do the presence of snow leopards benefit environment of Narphu?	14	20	31	−17	−0.26
4	Is it good to teach community about the snow leopards?	22	37	6	16	0.25
5	Where should snow leopards be protected?	21	20	24	6	0.09
6	Will you support ACAP in conserving snow leopards?	19	42	4	15	0.23
7	How will you react if a snow leopard kills your livestock?	9	28	28	−25	−0.38
	Total	29 (45%)	4 (6%)	32 (49%)	5	0.08

^1^ Probable answers for question 1, 2, 3, 4 and 6 (with scores): Yes (+1), Not sure (0) and No (−1). Similarly, probable answers for question 5: Inside and outside protected area (+2), only inside protected area (+1), Don’t know (0), Zoo (−1) and Nowhere (−2), and for question 7: Take it as normal (+2), Ask ACAP for compensation (+1), Nothing (0), Chase it away (−1) and Kill/poison it to prevent future predation (−2). QN = Question number.

## Supplementary Materials

The followings are available online at https://www.mdpi.com/2076-2615/10/11/2187/s1, File S1: Questionnaire form for interview, Table S1: Full models result from GLMMs for livestock loss due to snow leopard.
