# Supplementary material for: What Factors Predispose Households in Trans-Himalaya (Central Nepal) to Livestock Predation by Snow Leopards?
Source: Animals (Basel). 2020 Nov 23;10(11):2187. doi: 10.3390/ani10112187 (PMC7700291; doi:10.3390/ani10112187)
Supplement: Supplementary file 1 [file animals-10-02187-s001.pdf]

animals

MDPI

Article

What Factors Predispose Households in Trans-Himalaya (Central Nepal) to Livestock Predation by Snow Leopards?

Mahesh P. Tiwari, Bishnu P. Devkota, Rodney M. Jackson, Bir Bahadur Khanal Chhetri and Sistata Bagale

Supplementary Materials:

Table S1. Full results from GLMMs for livestock loss due to snow leopard. TL = total number of livestock owned, LS = number of large stocks owned, DP = days livestock housed in pasture, CN = number of corrals, FS = family size and EDU = education status of the households.

Model	df	logLik	AICc	delta	weight
TL × LS	6	-140.04	293.52	0.00	0.32
TL × LS + FS	7	-139.59	295.15	1.63	0.14
TL × LS + EDU	7	-139.97	295.90	2.37	0.10
TL × LS + CN	7	-140.04	296.04	2.51	0.09
TL × LS + DP	7	-140.04	296.04	2.51	0.09
TL	4	-143.82	296.32	2.79	0.08
TL + FS	5	-142.91	296.84	3.32	0.06
TL + LS	5	-143.31	297.63	4.11	0.04
TL + EDU	5	-143.77	298.57	5.04	0.03
TL × LS + DP	8	-140.03	298.64	5.12	0.02
TL + LS +DP	6	-142.75	298.95	5.43	0.02
TL + LS + DP + FS	7	-141.95	299.87	6.34	0.01
CN	4	-146.70	302.07	8.54	0.00
LS	4	-147.95	304.57	11.04	0.00
DP	4	-149.25	307.18	13.65	0.00
FS	4	-151.22	311.11	17.59	0.00
Intercept only	3	-154.66	315.71	22.19	0.00
EDU	4	-154.62	317.91	24.39	0.00

File S1

Questionnaire form for recording livestock loss and people's attitude towards snow leopard conservation

A. General information of respondents

1. Respondent Name: Age: Sex: [ ] Male [ ] Female
2. Family size: [Male: Female:]
3. Education stauts of respondent in years: …………..
4. Family member's education status in years:

5. Major income sources:
   a. Animal husbandry
   b. Yarshagumba/NTFP
   c. Remittance
   d. Agriculture

e. Service/job f. Hotel

6. Land holdings:

B. Status of livestock and herding practices

7. Livestock numbers and trends (Trends: 1 = increasing, 2 = decreasing, 3 = stable

SN	Livestock type	Number of livestock	Age	Number of insured livestock	Trends	Reason for trends

8. Please mention in detail the name of pastures/grazing areas where you herd/graze your livestock in different seasons. Also mention the tentative months of grazing/herding your livestock in the pastures.

	Summer (Jun-Aug)	Autumn (Sep-Nov)	Winter (Dec-Feb)	Spring (Mar-May)
Name of pastures				
Months/days of stay				
Reason for moving				

9. How do you herd your livestock?
   a. Keep herders b. Own self c. Herd in group

C. Livestock depredation

10. Please name the wild animals that are seen in your village/pasture/rangeland.
11. Have you ever seen a snow leopard in your area? a. Yes b. No
    If Yes, please mention where and when have you seen and how many times?
12. What is the trend of snow leopard in your area in last 2-3 years?
    a. Increasing b. Decreasing c. Stable d. Don't know
13. What is the trend of blue sheep in your area in last 2-3 years?
    a. Increasing b. Decreasing c. Stable d. Don't know
14. Please give the details of livestock lost over the past two years. Specify year, month, time (1=Morning, 2=Day, 3=Evening, 4=Night), location (pasture name or any specific location), place (1=corral, 2=pasture), circumstances (1=guard present, 2=guard not present) and causes (1=snow leopard, 2=disease, 3=natural cause, 4=accidents, 5=other carnivores, 6=starvation). \

Date	Location	Place	Livestock type	Age	Time	Causes	Circumstances

15. Why do you think snow leopard attack/kill livestock?
16. What is the trend of depredation due to snow leopards in last two years?
    a. Increasing b. Decreasing c. Stable d. Don't know
    If, increasing/decreasing, why?
17. What can be done to prevent livestock predation due to snow leopard?
    a. Graze in groups c. Avoid predation risk zone
    b. Careful herding practice d. Remove snow leopard
18. What are the measures adopted by you to prevent the livestock predation by snow leopard?
19. Where do you keep your livestock in house/pasture?
    a. Traditional corral b. Predator proof corral c. Free grazing in pasture
    Also, mention the number of corrals that you own. Among them how many are predator proof?
20. Have you ever heard of trapping/poisoning or killing or snow leopard in your area to reduce livestock predation due to snow leopards? a. Yes b. No

D. Compensation and insurance scheme

21. Have you heard about Government Wildlife Damage Compensation/Relief guideline for your lost livestock? a. Yes b. No
22. Are you satisfied with the above-mentioned scheme or its process or compensation amount? If No, what should be done to make it more effective?
23. Does your village have any community managed livestock insurance scheme? Who manages the scheme? Are you satisfied? How can it be improved?
24. Please give the details of compensation that you have received over past two years for your lost livestock.

Year	Livestock type	No of death/killed	Amount received	Compensation received from

E. Attitude towards snow leopard conservation

1. Do you always want to see snow leopard in Narphu valley?
   a. Yes (+1) b. Not sure (0) c. No (-1)
2. Do you think snow leopard should be conserved?
   a. Yes (+1) b. Not sure (0) c. No (-1)
3. Do the presence of snow leopard benefit environment of Narphu valley?
   a. Yes (+1) b. Not sure (0) c. No (-1)
4. Is it good to teach community about snow leopard?
   a. Yes (+1) b. Not sure (0) c. No (-1)
5. Where should snow leopard be protected?
   a. Inside and outside protected area (+2) d. Zoo (-1)
   b. Only in protected area (+1) e. Nowhere (-2)
   c. Don't know (0)
6. Will you support ACAP in conserving snow leopard?
   a. Yes (+1) b. Not sure (0) c. No (-1)
7. How will you react if snow leopard kills your livestock?

a. Take it as normal (+2)
b. Ask ACAP for compensation (+1)
c. Nothing (0)
d. Chase it away (-1)
e. Poison/kill it to prevent future predation (-2)

Any other information:
